# Psychosis and Spinal Chronic Pain – Our experience

**DOI:** 10.1192/j.eurpsy.2025.1891

**Published:** 2025-08-26

**Authors:** J. Đokić, L. Ilić, S. Momirović, I. Nikolić

**Affiliations:** 1 Special Psychiatric hospital “Dr Slavoljub Bakalović”, Vršac; 2 Clinic for Neurosurgery, University Clnical Center of Serbia; 3 Medical Faculty, University of Belgrade, Belgrade, Serbia

## Abstract

**Introduction:**

Most common pain in psychosis is headache. On the second place are spinal syndromes. Often patients have chronic spinal pain, and the treatment of neuropathic component is difficult because polymedication and possible drug interaction.

**Objectives:**

The aim of our study was the intersection of the state of therapy and therapeutic response in patients with spinal chronic pain and psychosis.

**Methods:**

This cross-sectional study includes 25 patients treated at the Department for woman chronic psychosis in the SPH “Slavoljub Bakalovic” in Vršac during their hospitalization. The covered period was from April 1^st^ to August 31^st^ 2024.

**Results:**

During our research, 60 female patients with psychosis were treated at our department, and 25 (41.67%) had spinal hronic pain. The average age of the patients was 60 years (41-75), and the duration of symptoms was from 6 months to 11 years (average 2 years and 2 months). Localization was mainly in the area of the lower back (12), cervicobrachialgia (7), lumboischialgia (5) and only in the lower extremities (1). According to the type of pain, all of them had predominantly neuropathic pain. The average value of pain intensity on the VAS scale was 4 (3.92). All patients were treated with non-steroidal analgesics and 16 of them had benzodiapines in therapy. Along with the mentioned therapy, 9 (36%) patients received a coanalgetic from the group of anticonvulsants and 5 (20%) from the group of antidepressants. Only 4 patients used during the hospitalization supplement based on vitamin B complex. A good therapeutic response was achieved in 20 (80%) patients (reduction of pain on the VAS scale by 2 or more points), partial in 1 patient (reduction of pain on the VAS scale by 1 point). In 4 patients, the prescribed therapy did not reduce pain.

**Conclusions:**

Chronic pain of spinal origin occurs in an approximate percentage as in the general population, but the problem of its treatment is the primary disease and polymedication in the therapy of the primary disease.With a well-balanced therapy, a good therapeutic response in pain reduction can be achieved.

**Disclosure of Interest:**

None Declared

